# A review of 40 years of enteric antimicrobial resistance research in Eastern Africa: what can be done better?

**DOI:** 10.1186/s13756-014-0041-4

**Published:** 2015-01-28

**Authors:** Sylvia Omulo, Samuel M Thumbi, M Kariuki Njenga, Douglas R Call

**Affiliations:** Paul G. Allen School for Global Animal Health, Washington State University, Pullman, WA USA; Kenya Medical Research Institute, Kisumu, Kenya

**Keywords:** Antimicrobial resistance, Eastern Africa, Minimum reporting guidelines

## Abstract

The emergence and persistence of antimicrobial resistance is driven by varied factors including the indiscriminate use of antibiotics and variable drug efficacy and presents a major threat to the control of infectious diseases. Despite the high burden of disease in sub-Saharan Africa and the potential health and economic consequences, the level of research on antimicrobial resistance in the region remains unknown. Little data exists to quantify the contribution of different factors to the current levels of antimicrobial resistance. To identify the factors that contribute most to the emergence, amplification, persistence and dissemination of antimicrobial resistance in humans and animals, we used the PRISMA 2009 guidelines to conduct a systematic review of studies on antibiotic-resistant enteric bacteria in Eastern Africa. We searched PubMed and Google Scholar databases and identified 2,155 probable articles, of which 89 studies on humans and 28 on animals remained after full-text review. These were articles from Kenya, Tanzania, Uganda, Ethiopia, Rwanda and Burundi, published between 1974 and 2013, that reported resistance in *Salmonella*, *Shigella*, *Escherichia coli* and *Vibrio* sp. The majority (98%) of human studies were based on hospital- (rather than community-wide) sampling and although they report high levels of antimicrobial resistance in the region, study design and methodological differences preclude conclusions about the magnitude and trends of antimicrobial resistance. To remedy this, we discuss and propose minimum reporting guidelines for the level of detail that should be explicitly provided for antimicrobial resistance study designs, testing of samples and reporting of results that would permit comparative inferences and enable meta-analyses. Further, we advocate for increased focus on community- rather than hospital-based sampling to provide a better indication of population-wide trends in antimicrobial resistance. This approach, together with the establishment of a robust regional surveillance network, should over time build a pool of evidence-based data useful for policy decisions and interventions aimed at controlling antimicrobial resistance.

**Electronic supplementary material:**

The online version of this article (doi:10.1186/s13756-014-0041-4) contains supplementary material, which is available to authorized users.

## Introduction

Since the discovery of penicillin in 1928, antibiotics and other antimicrobial therapies have been used to control both old and new emerging pathogens, resulting in global improvements in disease outcomes and increments in life expectancy [1,2]. However, the rapid emergence of antimicrobial resistance (AMR) by microbial pathogens threatens to reverse the public health gains made since widespread use of antibiotics was adopted. AMR is not a recent phenomenon, [2] and with decreasing options for- and production of newer antibiotics [3-6] the control of diseases has become a challenge, particularly in low- and middle-income countries where infectious diseases, poverty and malnutrition are endemic.

The emergence of AMR is a complex process often involving the interplay of human, environmental and pathogen-related factors [1,2,7,8]. In sub-Saharan Africa, the endemicity of acute respiratory infections, diarrheal diseases, HIV/AIDs, tuberculosis, malaria and helminthic infections has increased the demand for antimicrobial therapies both for prophylaxis and treatment. Further, shortfalls in the healthcare environment ranging from limited diagnostic capacity and resources, unregulated access to antibiotics, constrained access to health facilities and poor training with respect to antibiotic use [7-13] have increasingly stoked the demand for antibiotics. Veterinary use of antibiotics is also thought to contribute to antibiotic resistance in humans although little is known about how significant this contribution is in sub-Sahara Africa. Our ability to assess these contributions is limited largely by the absence of surveillance on antibiotic use both for therapeutics and prophylaxis. Unfortunately, only limited resources have been devoted to researching of this problem [10].

In 2011, the Global Antimicrobial Resistance Partnership (GARP) - Kenya Working Group stated: “without knowing the levels or trends of antibiotic resistance or how key actors are performing, it is impossible to make rational recommendations or monitor the effectiveness of interventions”. We therefore conducted a review of published work from countries in eastern Africa (Kenya, Uganda, Tanzania, Rwanda, Burundi and Ethiopia) to assess what can be learnt from published data on AMR in the region. We focused on antibiotic-resistant enteric bacteria because these represent the most immediate urgent global concern [5,6] and diarrheal diseases are among the most common causes of morbidity and mortality in low-income nations, disproportionately affecting children under the age of five [8,13]. Our goal was to critically analyze the progress of human and animal research in the region and discuss principles that are potentially useful to guide efforts aimed not only at controlling AMR in bacteria, but also in viruses, protozoa and fungi.

## Methods

Between October 2013 and March 2014, PubMed and Google Scholar databases were queried for articles containing the search terms presented in Table 1. A proportion of research articles from eastern Africa were published in journals that are not indexed in MEDLINE. Consequently, reference lists from identified articles were used to collate additional publications. Articles were selected for further evaluation based on the following inclusion criteria: (i) relevance to antimicrobial resistance in enteric bacteria, (ii) publication in English or French, and (iii) accessibility of the full-length article. For our purpose, selection was not limited by the year of publication; historical data was useful in informing us on the progress of AMR research in the study area.

**Table 1:** **Key search terms used in PubMed and Google scholar**

**Initial search terms**	**Refining terms**
“Antibiotic resistan*”	“east* Africa*”
“Antimicrobial* resistan*”	“east* Africa*countr*”
“Drug* resistan*”	“Kenya*”
“Multi-drug resistan*”	“Uganda*”
“Multidrug resistan*”	“Tanzania*”
“Multiple-drug resistan*”	“Ethiopia*”
“Multiple drug* resistan*”	“Rwanda*”
“Antibiotic* susceptib*”	“Burundi”
“Antimicrobial* susceptib*”	“enterobacteria*”
“Drug* susceptib*”	“enter* pathogen*”
“Multi-drug susceptib*”	“diarrh* pathogen*”
“Multidrug susceptib*”	“Salmonella* resistan*”
“Multiple-drug susceptib*”	“Shigella* resistan*”
“Multiple drug* susceptib*”	“*Vibrio* resistan*”
	“Escherichia* resistance*”

Initial search terms included words used to filter out publications that did not address antimicrobial resistance. Refining terms were then applied to select only articles from the study region and on the pathogens of interest. Truncation marks (*) indicate that different extensions of the main stem of words were used.

We reviewed the abstracts of all articles that met the above inclusion criteria. Where insufficient detail was provided, the entire article was reviewed before its inclusion or exclusion was determined. Duplicate references or publications reporting the same data in different journals were excluded. Most articles (82%) on antibiotic-resistant *E. coli* from humans were identified by scanning reference lists of selected publications. Other relevant articles were obtained through personal references and publications posted in curriculum vitae of some of the authors.

Additional general information was garnered from reports by the Alliance for the Prudent Use of Antibiotics [9,13], the Ecumenical Pharmaceutical Network [11], the World Health Organization [3-6] and the Global Antibiotic Resistance Partnership [10]. Remnant literature resulting from the search described above provided useful information on AMR in the greater African region.

### Data extraction for analysis

The fields considered for this review included author, year of publication, study duration, country, study setting (rural, urban), study design (hospital-, laboratory- or community-based), age demographics (children, adults) or studied animal, sample type collected, laboratory tests performed, laboratory standards used for interpretation of results, bacterium (genus) isolated, number of isolates obtained, number of resistant isolates and antibiotics tested. For analysis: (i) study “settings” were classified as ‘mixed’ for samples drawn from both rural and urban populations; peri-urban and urban studies were pooled; (ii) study “designs” were considered ‘hospital-based’ if exclusively conducted in a hospital laboratory as part of patient management or if they contained AMR data extracted from hospital/patient records; ‘laboratory-based’ if they retrospectively analysed stored samples or clinical samples that were not used for patient management; or ‘community-based’ if based on population-scale sampling irrespective of disease status; (iii) bacteria were collectively identified by their genus (except *E. coli*); and (iv) antibiotics with different trade names were identified using one name (thus cotrimoxazole included sulfamethoxazole/trimethoprim while augmentin included amoxicillin-clavulanic acid) or when authors used general classifications (as in the case of sulphonamides, which therefore included sulfisoxazole, sulphadiazine, sulfamethoxazole).

Given variation in study execution and reporting techniques, publications were carefully scrutinized when extracting data. Occasionally, this involved making the best possible judgments from the available data. When crucial information was required but lacking, authors were contacted (at least three times) via email to provide clarification. Eventually, such publications were either included or excluded depending on whether or not the required information was provided. Data extracted from included articles was summarized in Excel and SigmaPlot v11 · 5.

## Results

### Study selection

**Figure 1:**
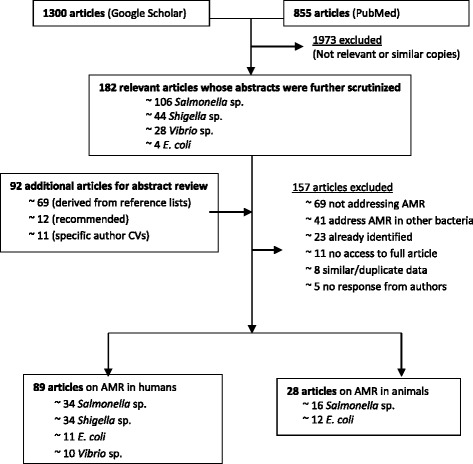
**Flow diagram summarizing the selection of publications for review.** Two exclusion steps were applied. Total articles excluded (underlined) and reasons for exclusion are shown. CV: *Curriculum vitae.*

**Figure 2:**
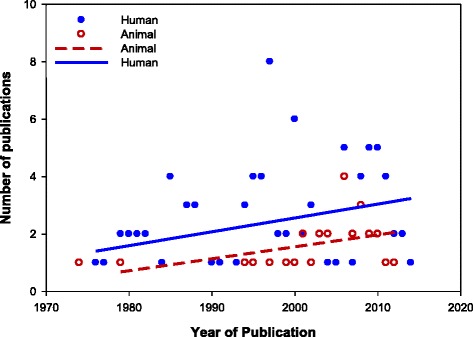
**Distribution of reviewed publications from 1974 to 2013.** Trend (based on year of publication) shown for human (blue full dots) and animal (red circles) studies from the six countries studied. Regression lines show an increasing trend in the number of publications from the mid-1970s to date.

Our search terms (Table 1) identified 2,155 probable articles. Of these, only 105 English and 12 French articles published between 1974 and 2013 met the criteria for inclusion (Figure 1). 89 were studies on humans and 28 on animals. One study [14] concurrently tested human and animal samples and was thus counted both as a human and animal study. Generally, the number of publications increased progressively from 1974 to 2013 (Figure 2).

### Study characteristics

#### Population

Of the 89 articles focused on research in humans, 66% were from Kenya and Ethiopia with those from Burundi, Rwanda, Tanzania and Uganda accounting for the remaining 34% (Table 2). Most of these were hospital-based (61%) or lab-based (37%) studies reporting cross-sectional, retrospective or outbreak-related AMR data. Only two studies were considered community-based. Isolates were more commonly cultured from persons of all ages (66%) than solely from adults (14%) or children (20%) and represented a fair distribution of both rural (30%) and urban (49%) settings; mixed settings accounted for the remaining 21%. Similar to human studies, animal studies were mainly from Kenya and Ethiopia. No animal studies were identified from Uganda, Rwanda and Burundi and only one study was identified from Tanzania (Table 2). These studies were predominantly community-based (93%) with samples drawn from cattle, chickens, pigs, camels, sheep, goats, rats and fish mostly in urban (75%) than rural (24%) or mixed settings (1%). Studies that reported resistant bacteria from animal products such as milk, meat and eggs were included in the analysis. Approximately half (53%) of animal studies focused on poultry or cattle.

**Table 2:** **Distribution of publications from the six countries studied shown by age of study subjects and by pathogen tested**

		**Bur**	**Eth**	**Ken**	**Rwa**	**Tan**	**Uga**		
**A. Human studies**								**Total**	**Citation**
***E. coli***	Adults	-	-	1	-	-	-	1	[15]
	Children	-	-	5	-	-	1	6	[14,16-20]
	All ages	-	1	2	-	-	1	4	[21-24]
	**(n)**	**0**	**2**	**8**	**0**	**0**	**2**	**11**	
***Salmonella*** **sp.**	Adults	-	4	5	-	-	-	9	[25-33]
	Children	-	2	5	-	1	1	9	[34-42]
	All ages	-	4	10	1		1	16	[43-58]
	**(n)**	**0**	**10**	**20**	**1**	**1**	**2**	**34**	
***Shigella*** **sp.**	Adults	-	2	-	-	-	-	2	[59,60]
	Children	-	2	-	-	1	-	3	[61-63]
	All ages	4	9	6	8	1	1	29	[64-91]
	**(n)**	**4**	**13**	**6**	**8**	**2**	**1**	**34**	
***Vibrio*** **sp.**	All ages	1	1	-	4	3	1	10	[92-101]
	**N**	**5**	**25**	**34**	**13**	**6**	**6**	**89**	
**B. Animal studies**									
***E. coli***		-	1	11	-	-	-	12	[14,102-112]
***Salmonella*** **sp.**		-	14	1	-	1	-	16	[113-128]
	**N**	**0**	**15**	**12**	**0**	**1**	**0**	**28**	

(n) is the total number of studies on a particular pathogen from each country; N is the cumulative number of human and animal studies from each country. Last column shows citations.

#### Laboratory methods

For all studies, testing against resistance to antibiotics was done through agar dilution, broth microdilution, tube dilution, disk diffusion, E-test, Sensititre- (automated) or VITEK-2 (semi-automated) tests. Disk diffusion was by far the most commonly used method, particularly in human studies (90% vs. 66% in animal studies). Regardless of the method employed, only 40% of human studies compared with 78% of animal studies reported using some form of standard interpretation guidelines. These included guidelines by the Clinical and Laboratory Standards Institute, [formerly the National Committee for Clinical Laboratory Standards (NCCLS)], British Society for Antimicrobial Chemotherapy, Comité de I'Antibiogramme de la Société Française de Microbiologie, Deutsches Institut für Normung (DIN) 58940, the Danish Integrated Antimicrobial resistance Monitoring and Research Program and the World Health Organization (WHO). Nevertheless, CLSI guidelines were the most commonly used (70% of human studies from Kenya and 67% of animal studies from Ethiopia). The use of reference strains for quality assurance was reported in 62% of human- and 56% of animal studies. *E. coli* ATCC 25922 was the most commonly identified standard isolate (human: 81% and animal: 87%).

#### Pathogens and resistances tested

Considering only one pathogen per study, *Salmonella* (38%) and *Shigella* (38%) were most commonly studied pathogens in humans, followed by *E. coli* (13%) and *Vibrio* sp. (11%) consecutively (Table 2). These proportions remained unchanged when 14 studies that concurrently tested two pathogens, four that tested three pathogens and two that evaluated all four pathogens were accounted for. In all, susceptibility results for these pathogens were reported for over 30 different antibiotics. However, for specific bacteria, *E. coli*, *Salmonella* and *Shigella* sp. isolates were most commonly (≥50% of studies) tested for resistance to ampicillin (Amp), chloramphenicol (Chl), ciprofloxacin (Cip), cotrimoxazole (Cot), gentamycin (Gen) and tetracycline (Tet) while *Vibrio* sp. for resistance to Amp, Chl and Tet. Animal studies on the other hand isolated either *Salmonella* (59%) or *E. coli* (41%), testing these mainly for resistance to Amp, Chl, Cot, Gen, kanamycin (Kan), Tet, nalidixic acid (Nal), streptomycin (Str), sulphonamides (Sul) and trimethoprim (Tri) [see Additional file 1]. For our purposes, comparisons between reported resistance levels were not performed given the large variability in reported variables and reporting styles.

In general, AMR in the region was reported to be increasing, presumably driven by multiple factors (Table 3). Importantly, while most authors made claims about the mechanisms that were likely to contribute to the observed AMR patterns, no studies were identified that actually investigated or quantified the contributory roles of any of these factors within the region.

**Table 3:** **Factors that explain the prevailing state of AMR in eastern Africa**

**I. Factors that favor the emergence, dissemination and/or persistence of AMR**	
**a) Factors common to human and animal studies**	
• Ease of access (cheap, widely available) to antibiotics	Kenya [19,32,36,51,123]; Uganda [22]; Ethiopia [30]; Tanzania [39,85]
• Antibiotic use practices, including self-medication, high frequency of antibiotic use, sub-therapeutic use or indiscriminate use	Kenya [19,26,31,32,36,51,54,77,108,129]; Ethiopia [27,47,88,89,120,125]; Tanzania [85,113]
**b) Human studies**	
• Over-prescription at health facilities due to limited diagnostics resources	Ethiopia [89]; Kenya [38]
• Severe infections requiring different antibiotics	Rwanda [79]
• Human importation of antibiotic resistant bacteria	Burundi [75]
• Nosocomial or community transmission of resistant bacteria	Kenya [14,36,78]; Rwanda [44]
**c) Animal studies**	
• Resistant bacteria imported via contaminated food	Kenya [26]; Ethiopia [122,125,126]
• Antibiotic use in humans	Kenya [105-107]; Ethiopia [122,125]
• Animal-animal contact	Ethiopia [119]
• Animal-human close co-existence increasing contact	Kenya [103]
• High antibiotic use in animals in small production systems, poor farm management practices disseminating resistant bacteria	Kenya [107]; Ethiopia [126]
• Housing contamination	Ethiopia [124]
• Contamination during handling animal products.	Kenya [107]; Ethiopia [115,117,125]
**II. Factors that contribute to the reduction of AMR**	
• High cost of antibiotic	Kenya [36]; Ethiopia [117,118]^Ɨ^
• Limiting antibiotic availability	Uganda [22]; Rwanda [79]; Ethiopia [118,119]^Ɨ^
• Periodic withdrawal of antibiotics from public use	Kenya [37]; Rwanda [79]
• Parenteral administration of antibiotics	Ethiopia [47]
• Infrequent or prudent use of antibiotics	Kenya [104,107,123]^Ɨ^; Ethiopia [115,117,120,125,127]^Ɨ^

List of risk factors that are thought to contribute to the state of antimicrobial resistance in Eastern Africa as suggested both by studies on AMR in humans and animals. Country and relevant citation shown in the column on the right. ^Ɨ^Animal studies.

## Discussion

The goal of this review was to assess the current knowledge of AMR for enteric bacteria found in eastern Africa. Specifically, we set out to understand the contribution of different factors to the emergence, amplification, persistence and dissemination of antibiotic resistance for both human and animal populations. After collating the data and conducting exploratory analyses, we found it difficult to make meaningful comparisons from studies due to the differences in study designs and styles of reporting of results. Here we provide a general view of the progress made in AMR research while highlighting gaps that impede our understanding of the dynamics of AMR in eastern Africa. We also propose potential ways to address these gaps to improve the quality of AMR data and build a pool of evidence-based data for this region. These are likely to improve our understanding of the mechanisms that contribute most to AMR, the regional prevalence and the trends of AMR in the short- or long-term.

### Trend of AMR research in eastern Africa

The gradual increase in publications from the mid-70s to date suggests that AMR research is gaining increasing attention within eastern Africa (Figure 2). While most of the reported AMR research was conducted in Kenya and Ethiopia, we also observed an increase in AMR research in Uganda and Tanzania, although fewer publications were identified from these countries (Table 2). It is possible that researchers from these countries have focused their AMR research on non-enteric pathogens such as *Mycobacterium tuberculosis*, which were not considered in this review. We found very limited data from Rwanda and Burundi after 1995, perhaps owing to historical political events that could have disrupted health-related surveillance or research studies if these existed. We, however, attempted to gather such data by including reported studies carried out on these citizens in refugee camps of neighboring countries [96].

Notably, even in countries from which more publications were derived, research progress on AMR in enteric bacteria appears slow relative to the global awareness of AMR, supporting the seemingly low prioritization of this problem in sub-Saharan Africa [10]. This is worrisome considering that a sizable portion of health budgets in these countries are allocated to the acquisition of antibiotics for the prevention or treatment of infectious diseases, including diarrhea [9]. Further, the consistently fewer publications on animal studies over time indicate a biased focus on AMR research in humans. Justifiably, human diseases are a primary concern, particularly when those affected are the most economically productive sub-populations. Nevertheless, in the case of zoonotic food and water-borne diseases, control of human disease relies, in part, on the control of animal diseases. With projected increases in human and food animal populations in the coming decades, increased interactions between humans and animals are inevitable, particularly where land for expanding populations is scarce. Presumably, animals can also serve as reservoirs of antibiotic resistant enteric bacteria, underscoring the importance of integrating animal and human research to maximize benefits for both sectors (i.e., a One Health focus).

Based on our review, research in eastern Africa has been focused on AMR prevalence and patterns; a trend that has persisted since the 1970s. The frequency of studied bacteria (*Salmonella*, *Shigella* in humans and *Salmonella* and *E. coli*) corresponds with the frequency of their implication in diarrheal diseases in the region, their potential for zoonotic transmission to humans and their high rates of resistance to available treatment regimens (Amp, Chl, Cip, Cot, Gen, Kan and Tet). Probable drivers and potential mitigation actions were universally discussed by study authors. Nevertheless, no study directly tested these ideas or assessed the effectiveness of AMR interventions. Similarly, none of the studies tested associations between these putative risk factors and reported prevalence of AMR; consequently, although mechanistic explanations were suggested and may be intuitively reasonable, they remain speculative. While the potential role of risk factors such as antibiotic use is undisputed, a consistent focus on cross-sectional prevalence data does not build our understanding of the proportional contributions and distributions of each of these factors in different environments and subpopulations. Thus, while useful for qualitative purposes, unstructured and uncoordinated prevalence data is insufficient for estimating changes over time and for designing focused interventions. We submit that the following issues present the greatest challenges to drawing inferences from the existing research:

#### Study execution

##### Study and sampling design

Of the three study designs (hospital-, laboratory- and community-based), AMR studies involving humans were predominantly hospital- or laboratory-based. Samples and isolates for these studies were obtained primarily from patients seeking treatment at health facilities and, in general, reported high prevalence of AMR. While hospital sampling is more convenient and less expensive than field-level random sampling, it likely represents populations that - owing to failures in self-medication with variable-quality antibiotics - are pre-selected for resistant strains of bacteria thereby inflating reports of AMR prevalence. Similarly, where the hospital environment facilitates infection transmission, as in the case of non-typhoidal *Salmonella* (NTS), [26,130] hospital and community prevalence may differ, particularly in communities that rely on non-antibiotic forms of therapy [23]. Studies that have reported the occurrence of AMR in populations not previously exposed to antibiotics [131-136] or reported unchanged AMR prevalence despite frequent- [137-139] or infrequent-[140] antibiotic exposure, indicate that the prevalence of- and drivers for AMR may vary. Consequently, while hospital samples provide an important means of characterizing AMR, their generalizability to the general population is limited. Randomized and independent sampling, akin to methods employed in community-based studies, need to be considered as the basis for future sampling efforts. Animal studies, though community-based, generated limited data after grouping by relevant variables, thereby limiting our ability to delineate patterns or draw comparisons between countries over time.

##### Laboratory protocols

Different laboratory assays were used for antibiotic resistance testing, with automated systems coming into use after the year 2000. Occasionally, modifications to these assays were used such as single- or double-disc diffusion, controlled agar diffusion and gradient agar diffusion (E-test), and instances occurred where two tests were employed. This was either done in combination (to simultaneously determine antibiotic sensitivity and minimum inhibitory concentrations (MICs), or when testing was done in different laboratories) or separately (each test for a specific set of antibiotics as was the case where certain antibiotics were not included in automated systems). Where combined testing was reported, however, it was unclear how disparities between tests were resolved in the event that this occurred, or which of the test results were reported (if done in different labs). Importantly, most studies reported the use of a reference strain or a standard, particularly *E. coli* American Type Culture Collection (ATCC) 25922, with some studies reporting use of up to six different reference strains. Interpretation standards were equally varied although most studies that reported use of a guideline after 1997 reported using the CLSI guidelines recommended by the WHO. There were, however, reported modifications of the common laboratory assays and it was unclear how these were standardized to ensure agreement between tests.

#### Non-standardized reporting

We noted large variability in the scope of reported data and in some cases limited detail on the description of study methods and results. This could have been due to page limitations imposed by specific journals and/or the absence of a structured reporting system for AMR research. Most of the gaps in our data arose from inadequate description of (i) study period (date and duration), (ii) population demographics, and (iii) laboratory procedures (isolation techniques, controls and standards). These elements, discussed below, may be critical in detecting subtle yet significant differences between populations, procedures and time points, differences that may otherwise not be appreciated when AMR data is considered generally.

##### Study period

**Figure 3:**
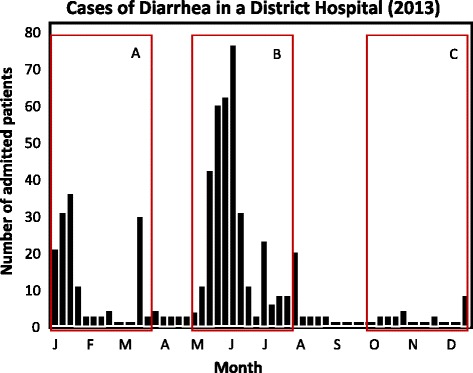
**Hypothetical cases of diarrhea in a district hospital in 2013.** Graph illustrating sources of potential differences in reported AMR prevalence arising from monthly variations in disease incidence reported in a hospital. **A**, **B** and **C** represent different sampling periods.

Depending on the nature and duration of a study, events (both natural and man-made) can intervene during the course of a study period to skew prevalence data in either direction. For instance, outbreaks of enteric diseases, commonly observed during floods or drought can increase health facility attendance and/or antibiotic usage thereby amplifying AMR prevalence during such periods. As a hypothetical example, consider cross-sectional studies conducted over period A, B and C (Figure 3). All three occur in the same hospital but ultimately yield different data from each other. Providing ‘time data’ while identifying factors associated with AMR during study periods thus becomes crucial in explaining patterns or deviations that would otherwise be interpreted incorrectly.

##### Population demographics

Factors such as age, gender, ethnicity, environment and health status can limit the generalizability of AMR data. Children, who often are at a higher risk of diarrhea and other infections, are likely greater consumers of certain types of antibiotics than others and could contribute more to the AMR prevalence for some antibiotics as compared to adults. In animal studies, calves have been shown to harbor more antibiotic resistant bacteria than adult cattle [139]. Gender roles, on the other hand, can have an indirect bearing on AMR by affecting health-seeking behavior. For instance, health-services utilization by men can be lower than among women whose child-rearing roles present opportunities for seeking treatment particularly when a child is sick. Cultural traditions and practices can also explain differences in AMR levels and profiles. A study conducted among Maasai in Kenya reported lower AMR prevalence than that from other parts of the country possibly owing to their practice of traditional medicine [23], although confounding factors may exist in such studies. For pastoralist communities (e.g. the Maasai in Tanzania) where antibiotic use in animals is common (Call *et al.* unpub. data), common AMR profiles in human and animal bacteria may be useful in identifying and studying AMR zoonotic transmission pathways.

AMR prevalence can also vary by study setting. For instance, Kariuki *et al.* [36,37] found a significantly higher prevalence of NTS in children from informal settlements (slums) than those from higher socio-economic classes. These children, who were underrepresented in hospital populations were also the majority bearers of invasive NTS, consistent with socioeconomic barriers that limit their presentation to health facilities in the event of failure of self-medication. Similarly, rural populations may have, among other differences, poorer sanitary conditions, greater human-animal interactions, limited access to treatment facilities and fewer varieties of effective antibiotics [6,141,142], all which can impact their AMR prevalence and profiles when compared to urban populations.

##### Laboratory procedures

Subtle variation in laboratory protocols can impact the interpretation of antibiotic sensitivity results [143]. There are multiple steps involved in quantifying antibiotic susceptibility/resistance, and consequently multiple potential sources of variation among studies that can impact the validity of AMR data. Some of the parameters that have been tested in this regard include the size of the inoculum (i.e. number of cells), [143-147] growth media used, duration of incubation and incubation temperature [7,145,146], inoculum dispensing systems [146,148], delays in incubation following disc application, depth of medium, spacing of discs, potency of antibiotic discs, media composition and pH, and subjectivity of ocular readings [7,143,145]. These factors underscore the importance of standardized testing and quality control that are needed to improve and maintain the validity of AMR data; the adoption of which is still low in the region, especially in human AMR research studies.

The individual and collective contribution of each of these factors cannot be appreciated fully in the absence of guidelines that ensure consistent reporting of such variables. We propose a means for incorporating this data when reporting AMR data (see Appendix). The opportunities to implement a structured AMR surveillance system are probably limited for many low-income countries owing to competing national priorities and scarcity of resources. Nevertheless, it is still feasible for scientists in these regions to adopt a structured reporting mechanism for AMR studies so that the data collected can be used to make meaningful comparisons between different studies, geographic locations and points in time. Given widespread adoption, such guidelines should make it possible to compile AMR trends, highlighting variation between regions and guiding the implementation of focused interventions based on data from what would otherwise be scattered amongst reports. The potential benefits of such a venture stand to be appreciated by research groups and public health policy-makers in the region and beyond.

## Conclusion

There is a growing body of literature describing AMR in sub-Saharan Africa and these studies are useful for identifying the kinds of resistance that are present in the region. Unfortunately, the focus on non-random samples and potentially pre-selected flora combined with a very diverse array of methodologies make it impossible to estimate trends in prevalence and incidence from this body of literature. Even less is known in animal populations. We contend that at minimum, a more structured reporting strategy is needed to aid future efforts in this regard. Ultimately, however, a significant investment is needed to develop a structured and rigorous region-wide antibiotic resistance surveillance network [6]. In the interim, our understanding of the AMR challenge in sub-Saharan Africa can substantially be improved by moving beyond descriptive studies to hypothesis-based projects that evaluate intervention strategies. Emphasis on quantitative assessment of risk factors rather than simply making assumptions on how AMR is influenced in study populations would be extremely valuable because inquiries such as these will inform policy far better than accumulation of even more descriptive and incomparable AMR studies.

## Appendix: Proposed Minimum Reporting Guidelines for Research on Antimicrobial Resistance

See Additional file 2 for detailed rationale and definitions for these guidelines. The following details should be provided when reporting results for antimicrobial resistance research:

### Study Structure

#### Design

Mention the type of study and provide a description of its design.

#### Dates

Indicate the months and years of the study and provide data on any potential confounding events (e.g., natural disasters) during the study period.

#### Duration

State the period of time during which samples were specifically collected.

#### Setting

Describe the sampling area specifying whether rural or urban. Studies in mixed settings, e.g., referral hospitals, should provide an approximate (in %) distribution of the catchment population (urban vs rural).

#### Population

Give the characteristics of the chosen population including the distribution by age (% children vs. % adults) and sex, and indicate all categories considered (e.g., cases vs. controls, HIV+/−, in- vs out-patient) that could influence AMR testing data.

#### Sampling

- Provide the criteria used for enrollment in the study.
- Indicate the types of specimen collected and how these were collected.
- Give the overall and specific numbers of samples collected (by category sampled).
- Indicate how samples were processed before storage.

### Testing Procedures

#### Samples

- Describe how specimens were handled, transported and stored after collection.
- State duration of sample storage before testing.
- Provide the number of samples tested and indicate reasons for exclusion, if any.

#### Reagents

- Indicate brands of commercial media, controls or antibiotic testing kits used.
- Describe how commercial and/or in-house reagents were prepared prior to testing.
- Indicate the validation methods used stating cut-off values applied.

#### Isolation

- Describe the method used to obtain isolates indicating:
  i. Incubation period and temperature.
  ii. Distinguish between technical and independent replicates
  iii. Methods used to identify/speciate isolates.
- State the overall number of isolates obtained per independent biological sample and provide an explicit description of how these were treated in the analyses.
- Specify the distribution of isolated organisms:
  i. By source (e.g. blood, stool, pus).
  ii. By category including sex and age. Note that age is particularly important because the prevalence of AMR bacteria can vary by age (greater in younger hosts).

#### Susceptibility/resistance testing

- Indicate the total number of isolates tested.
  i. Indicate the antibiotics tested providing antibiotic name and acronym, concentrations used, and interpretation cut-off values that were used.
- Describe the method(s) used to test sensitivity/resistance indicating:
  i. Type of assay used (disc diffusion, MIC, breakpoint).
  ii. Size of inoculum (McFarland’s units).
  iii. Number of independent and technical replicates included.
  iv. Reference strain used as a positive control standard.
  v. Incubation temperature and period.
  vi. Describe how the results were quantified:
    1. Zone size (mm) with X independent discs
    2. MIC read by ocular estimate or using plate reader?
    3. Breakpoint [yes/no]; were small colonies scored as present?
  vii. Describe how discordant results were resolved when more than one method was employed.
- Specify the testing standard used (e.g. CLSI, DIN, DANMAP)
- Indicate quality testing procedures were used (e.g. confirmatory testing by independent lab) and concordance level

### Results

i. Calculate the frequency of resistance to an antibiotic as the total number of resistant isolates divided by the total number of isolates tested with a given antibiotic.
ii. Tabulate resistances for ALL antibiotics tested indicating the absolute numbers or resistant isolates, and the percent (%) of resistant isolates.
iii. Stratify results by:
  - Pathogenic species, then by
  - Source of isolate, then by
  - Age groups and gender, then by
  - Other categories used
iv. Compare categories using appropriate statistics (contingency tests, ANOVA, etc.) with an explicit description of how replicates were defined and how technical vs. independent replicates were processed (this is important to avoid issues of pseudo-replication).

## Additional files

Additional file 1:
**Distribution of studies reporting antibiotic susceptibility for specific pathogens.**
Additional file 2:
**Rationale for the proposed guidelines and a description/definition of terms.**
