# Supplementary material for: A review of 40 years of enteric antimicrobial resistance research in Eastern Africa: what can be done better?
Source: Antimicrob Resist Infect Control. 2015 Jan 28;4:1. doi: 10.1186/s13756-014-0041-4 (PMC4339253; doi:10.1186/s13756-014-0041-4)
Supplement: Additional file 2: — Rationale for the proposed guidelines and a description/definition of terms. [file 13756_2014_41_MOESM2_ESM.docx]

**Supplementary information for use with the proposed guidelines**

**Rationale of the guidelines**

Implementation of a structured AMR surveillance system is limited for many low-income countries owing to competing national priorities and scarcity of resources. Nevertheless, it is feasible to adopt a structured reporting mechanism for AMR studies that can serve to standardize research reporting in a manner that allows meaningful comparisons between different studies, geographic locations and points in time. Given widespread adoption, such guidelines should make it possible to compile AMR trends, highlighting variation between regions and guiding the implementation of focused interventions based on data from what would otherwise be scattered reports. The potential benefits of such a venture stand to be appreciated by research groups and public health policy-makers in the region and beyond.

**Intended use**

Based on our literature review, we identified frequently encountered obstacles to making comparative inferences and conducting meta-analyses. To reduce these obstacles we recommend that, at a minimum, authors should provide the following information in an explicit manner. This guidance does not, in any way, prescribe the manner in which AMR research should be conducted. Rather, it serves to guide the researcher regarding the minimum level of detail that should be incorporated into their published findings in order to have the important benefit of extending the immediate and future impact of their research.

**Acronyms**

AMR Antimicrobial resistance

ANOVA Analysis of variance

CLSI Clinical and Laboratory Standards Institute

DIN Deutsches Institut für Normung

DANMAP Danish Integrated Antimicrobial Resistance Monitoring and Research Programme

HIV Human immunodeficiency virus

MIC Minimum inhibitory concentration

**Definitions and explanations**

For the purpose of these requirements, the following definitions apply.

**Adult** Any persons aged 18 and above.

**AMR research** Any study, regardless of design, that partially or wholly involves testing of susceptibility or resistance of microbes to (an) antimicrobial(s) with the intention of reporting levels of resistance to the antimicrobial(s).

**Child** Any person below the age of 18.

**Replicates** Assay replicates can be termed ‘technical’ or ‘biological’ as follows:

1. **Technical replicates** are assays results from the same sample/isolate;
2. **Independent replicates** are assay results from independent samples collected from different individuals.

**Study designs**  Every study needs an explicit description for how individuals (humans or animals) were selected for inclusion in the study. Where possible, randomization of sample selection should be done to limit study bias. It is crucial to inform the reader and future analyst about potential selection biases that may limit the ability to extrapolate findings beyond a given subpopulation.

1. **Case studies** - e.g., case reports and case series
   1. **Case report** - A detailed report of the diagnosis, treatment, and follow-up of an individual patient. Case reports also contain some demographic information about the patient (for example, age, gender, ethnic origin). [1]
   2. **Case series** - A group or series of case reports involving patients who were given similar treatment. Reports of case series usually contain detailed information about the individual patients. This includes demographic information (for example, age, gender, ethnic origin) and information on diagnosis, treatment, response to treatment, and follow-up after treatment. [1]
2. **Observational studies** – Studies in which individuals are observed or certain outcomes are measured. No attempt is made to affect the outcome (for example, no treatment is given). [1]
   1. **Case-control** – Comparison of a group of people with a disease or condition and a control group of people free from that disease. [2]
   2. **Cohort study** - A systematic study of a group of people which may be conducted prospectively or retrospectively. [2]
      1. A prospective cohort study involves a systematic follow-up for a defined period of time or until the occurrence of an event (e.g. onset of illness) in order to observe patterns of disease and/or cause of death.
      2. A retrospective cohort study examines data relating to the group’s history of exposure and disease experience.
   3. **Cross-sectional study** – The collection and analysis of information relating to persons in a population or group at a defined point in time (or defined period), with particular reference to their disease status, individual characteristics, and exposure to factors likely to predispose them to disease. [2]
3. **Experimental studies –** Studies in which the investigator intentionally alters one or more factors and controls the other study conditions in order to analyze the effects of the alteration. [3]
   1. **Clinical study/trial** - A type of research study that tests how well new approaches work in people. These studies test new methods of screening, prevention, diagnosis, or treatment of a disease. [1]
   2. **Randomized clinical trial** - A study in which the participants are assigned by chance to separate groups that compare different treatments; neither the researchers nor the participants can choose which group. [1]
4. **Population study** - A study of a group of individuals taken from the general population who share a common characteristic, such as age, sex, or health condition. This group may be studied for different reasons, such as their response to a drug or risk of getting a disease. [1]
5. **Survey** - An investigation in which information is systematically collected but the experimental method is not used. [3]
6. **Surveillance study** – The on-going systematic collection, analysis and interpretation of data essential to the planning, implementation and evaluation of public health practice, closely integrated with the timely dissemination of data to relevant stakeholders. [1]
   1. Active surveillance involves actively looking for cases.
   2. Passive surveillance involves gathering data through e.g. reported cases, documents.
7. **Uncontrolled study** - A study that lacks a comparison (i.e., a control) group. [1]

**Study setting Rural** if characteristic of the countryside; **Urban** if characteristic of a city or town [2]

**References:**

1. National Cancer Institute: NCI Dictionary of Cancer Terms. http://www.cancer.gov/dictionary. Accessed 5 May 2014.

2. Stevenson A, Lindberg CA (Eds): New Oxford American Dictionary. 3rd edition. New York: Oxford University Press; 2010.

3. Porta E (Ed): A Dictionary of Epidemiology. 5th edition. New York: Oxford University Press; 2008.
